# Evaluation of In Vitro Solar Protection Factor (SPF), Antioxidant Activity, and Cell Viability of Mixed Vegetable Extracts from *Dirmophandra mollis* Benth, *Ginkgo biloba* L., *Ruta graveolens* L., and *Vitis vinífera* L.

**DOI:** 10.3390/plants8110453

**Published:** 2019-10-26

**Authors:** Letícia Caramori Cefali, Janaina Artem Ataide, Ana Rita Fernandes, Elena Sanchez-Lopez, Ilza Maria de Oliveira Sousa, Mariana Cecchetto Figueiredo, Ana Lucia Tasca Gois Ruiz, Mary Ann Foglio, Priscila Gava Mazzola, Eliana Barbosa Souto

**Affiliations:** 1 Department of Pharmaceutical Technology, Faculty of Pharmacy, University of Coimbra (UC), 3000-548 Coimbra, Portugal; letisc82@yahoo.com.br (L.C.C.); janaina.a.ataide@gmail.com (J.A.A.); anaritavfernandes@gmail.com (A.R.F.); esanchezlopez@ub.edu (E.S.-L.); 2 Institute of Biology, University of Campinas (UNICAMP). R. Monteiro Lobato, 255, Campinas, Sao Paulo 13083-862, Brazil; 3 Faculty of Pharmaceutical Sciences, University of Campinas (UNICAMP), Rua Cândido Portinari, 200, Campinas, Sao Paulo 13083-871, Brazil; ana.ruiz@fcf.unicamp.br (A.L.T.G.R.); maryann.foglio@fcf.unicamp.br (M.A.F.); 4 Department of Pharmacy, Pharmaceutical Technology and Physical Chemistry, Faculty of Pharmacy and Institute of Nanoscience and Nanotechnology (IN2UB), University of Barcelona, 08028 Barcelona, Spain; 5 Networking Research Centre of Neurodegenerative Disease (CIBERNED), Instituto de Salud Juan Carlos III, 28049 Madrid, Spain; 6 School of Medical Sciences, University of Campinas (UNICAMP). R. Sergio Buarque de Holanda, 250, Campinas, Sao Paulo 13083-859, Brazil; ilzamo.sousa@gmail.com (I.M.d.O.S.); marianacecchetto@gmail.com (M.C.F.); 7 CEB—Centre of Biological Engineering, University of Minho, Campus de Gualtar, 4710-057 Braga, Portugal

**Keywords:** antioxidant, cell viability, flavonoids, quercetin, rutin, sun protection factor

## Abstract

The aim of this study was to validate a HPLC method for the assay of flavonoids in extracts obtained from natural sources, i.e., from *Dirmophandra mollis* Benth, *Ginkgo biloba* L., *Ruta graveolens* L., and *Vitis vinífera* L. The potential sun protecting effect, antioxidant activity, and cell viability of the extracts were also determined. Individual extracts (obtained from each individual species) and a mixed extract (containing the four extracts) were analyzed by the validated HPLC method for the identification of flavonoids and quantification of rutin and quercetin. An in vitro cell viability study was carried out using the neutral red method. The in vitro sun protection factor was determined by spectral transmittance and in vitro antioxidant efficacy was evaluated against DPPH, ABTS, and AAPH radicals. The HPLC method used for the identification and quantification of flavonoids in extracts exhibited linearity, precision, accuracy, and robustness. Detection and quantification limits were, respectively, 2.881 ± 0.9 μg·mL^−1^ and 0.864 ± 0.9 μg·mL^−1^ for quercetin, and 30.09 ± 1 μg·mL^−1^ and 9.027 ± 1.1 μg·mL^−1^ for rutin. All extracts did not affect cell viability at the evaluated concentration range and exhibited a sun protection effect and antioxidant activity. Among the evaluated extracts, *Ginkgo biloba* L. and the mixed extract depicted the most expressive antioxidant activity. The mixed extract exhibited sunscreen protection against ultraviolet A (UVA) and ultraviolet B (UVB) and a critical wavelength of 372.7 ± 0.1. Our results translate the enhanced flavonoids’ composition of the mixed extract, which may be a potential alternative over sunscreens and antioxidants in pharmaceutic/cosmetic formulations.

## 1. Introduction

Because of its many benefits, sun is essential to life, but sun radiation, especially ultraviolet A (UVA) and ultraviolet B (UVB), can stimulate free radical production, damaging mitochondrial enzymes and plasmatic membranes and causing reduction of antioxidant substances in the skin [1,2,3,4]. Moreover, UVA and UVB radiations can directly promote DNA damage, which is associated with skin aging acceleration and skin cancer, in addition to skin sunburns [5,6].

World Health Organization epidemiological studies show that ultraviolet (UV) radiation exposition, especially during childhood and adolescence, is the main etiological agent of skin cancer [7]. In Brazil, skin cancer is the most prevalent in the Brazilian population, which can be avoided by protection against sunlight, such as sunscreen use, stimulated by health education of the population [8].

Sunscreens are topical products initially developed to prevent sunburns, but have further evolved to protect against other harmful effects of ultraviolet radiation, such as skin cancer, aging, wrinkle formation, undesired pigmentation, and collagen loss [9,10]. Ideal UV filters should be non-toxic, not cause allergic reactions, and not be systemically absorbed [11]. Unfortunately, some concerns still remain about the safety of UV filters, as adverse effects, including contact sensitivity, vitamin D deficiency, allergic reactions, and photogenotoxicity, have been reported [12,13]. Moreover, there are also concerns regarding the accumulation of UV filters in the environment and potential risks of this accumulation [14]. Therefore, research on the use of natural ingredients aiming at reduction of skin irritation and other harmful effects associated with sunscreens is steadily increasing [15].

Among many plant compounds, flavonoids are the most studied in use as sun filters by presenting cyclic and aromatic rings in their structure absorbing in the ultraviolet region, especially at wavelength ranges of 240–285 nm and 300–550 nm [16]. Flavonoids are the third largest class of natural products and exhibit many important effects on plants, mainly regarding protection against pathogens and UVB radiation [17]. Rutin and quercetin are amongst the most common flavonoids and both have been evaluated for many different biological effects [18,19,20,21].

Continuing efforts in finding new sunscreens from natural products [22,23,24,25], the present study aimed at evaluating extracts of *Dimorphandra mollis* Benth, *Ginkgo biloba* L., *Ruta graveolens* L., and *Vitis vinifera* L., alone and as a mixture, as sunscreen filters using in vitro models.

## 2. Materials and Methods

### 2.1. Material

*Benitaka* grape (*Vitis vinifera* L. fruits) was purchased in single batch in a local supermarket. *Dimorphandra mollis* Beth fava beans were harvested in Private Biological Reserve (22°18′S/47°11′W), in Mogi Guaçu (São Paulo, Brazil), in May 2015. *Ruta graveolens* leaves were harvested in the Chemical, Biological and Agricultural Pluridisciplinary Research Center—CPQBA (22°48′S/47°0′W) at the University of Campinas (UNICAMP) (Campinas, São Paulo, Brazil) in April 2015. *Ginkgo biloba* L. dry extract was purchased at Galena (Campinas, São Paulo, Brazil). Ethyl alcohol, acetic acid, hydrochloric acid, boric acid, oxalic acid, acetone, ether, metallic magnesium, metallic zinc, ferric chloride, and aluminum chloride were provided by Synth (São Paulo, Brazil), and 1,1-diphenyl-2-picrylhydrazyl (DPPH), 2-2′-azino-bis(3-ethylbenzothiazoline-6-sulphonic acid (ABTS), 2,2′-azobis (2-amidinopropane) dihydrochloride (AAPH), and fluorescein by Sigma-Aldrich (São Paulo, Brazil). Quercetin (93.3% purity) and rutin (97.3% purity) standard by Acros (Sao Paulo, Brazil). RPMI-1640 and fetal bovine serum were provided by Gibco (Sao Paulo, Brazil) and Tris [hydroxymethyl] aminomethane and neutral red dye was provided by Sigma-Aldrich (São Paulo, Brazil).

### 2.2. Flavonoid Extraction

Fresh *Dimorphandra mollis* Benth fava beans and *Ruta graveolens* L leaves were dried in a stove (Lemaq, Mod LM-EST, Diadema, Brazil) at 40 ± 3 °C for 72 h, and then ground in hammer mill [26]. Fresh *Benitaka* grapes were analytically weighed, washed with neutral detergent and water, peeled, and grouped peels were dried at 40 ± 2 °C in a stove (Lemaq, Mod LM-EST, Diadema, Brazil) for 72 h. After weight, dry peels were ground in a blender (Black&Decker, Campinas, Brazil). For each species, extracts were obtained using ethanol at 1:3 (w:v) (Synth) in a liquid extractor by mechanic stirring at 27 ± 3 °C for 90 min. Thereafter, all extracts were subjected to vacuum filtration, solvent elimination (Marconi, MA120, Piracicaba, Brazil) at 40 ± 2 °C, and freeze-dried (Thermo Scientific, Power Dry PL3000, São Paulo, Brazil) [27]. All dry extracts, including commercial *Ginkgo biloba* leaf extract, were subjected to physical–chemical analysis such as pH, density, granulometry measurement, dry loss, total ash, and insoluble ash [28].

### 2.3. Flavonoid Identification and Quantification

All extracts were subjected to identification reactions [28], such as Shinoda, Taubock, Pew, Ferric chloride, and aluminum chloride for flavonoid identification.

### 2.4. Flavonoid Analysis by HPLC

#### 2.4.1. Sample Preparation

Dry extracts were resuspended in HPLC grade methanol (Merck) to achieve the final concentrations of 5 μg·mL^−1^ to *Dimorphandra mollis* Benth, 5 μg·mL^−1^ to *Ginkgo biloba* L., 50 μg·mL^−1^ to *Ruta graveolens* L., and 1000 μg·mL^−1^ to *Vitis vinífera* L. A mixed sample in 1:1:1:1 volume proportion was prepared containing the four extracts. Then, all samples were separately filtered in 0.45 µm membrane (Merck). Quercetin (50 μg·mL^−1^) and rutin (500 μg·mL^−1^) standard were analyzed by HPLC in comparison to extracts results.

#### 2.4.2. HPLC Conditions

Aliquots of 5 µL of each sample were injected in a HPLC–DAD instrument (Agilent, Technologies 1250 infinity), using monomeric chromatographic column C_18_ (Phenomenex), flow rate of 0.3 mL·min^−1^ for 10 min. The mobile phase used was methanol grade HPLC acidified with 0.1% (v/v) formic acid (Synth), at 27 ± 1 °C, and flavonoids quercetin and rutin were identified at 257 nm [29].

#### 2.4.3. Validation of Analytical Method by HPLC

The analytical method was validated according to parameters required by Brazilian Resolution nº 899 [30] for phytotherapeutic agents [31]. Analytical curves were obtained using concentrations of 50.0, 25.0, 12.5, 6.25, and 3.125 μg·mL^−1^ for quercetin and 500.0, 250.0, 125.0, 62.5, and 31.25 μg·mL^−1^ for rutin, both registered at 257 nm.

The precision assay was performed using intra-day and inter-day repeatability [30,31]. Six samples (analytical standard) with a concentration of 50 μg·mL^−1^ to quercetin and 500 μg·mL^−1^ to rutin were analyzed on the same day and on two consecutive days. Areas of the standards’ peaks were obtained and variation coefficient percentage was calculated. The mixed sample was also subjected to precision assay for reliability of results.

The accuracy test was performed through the recovery assay, which consisted of adding a known concentration of flavonoid standards to extracts. Then, different volumes (0.75, 1.25, and 1.75 mL) of quercetin (50 μg·mL^−1^) and rutin (500 μg·mL^−1^) were transferred to 5 ml volumetric flasks containing 0.625 mL of *Dimorphandra mollis* Benth (1 mg·mL^−1^), *Ginkgo biloba* L. (1 mg·mL^−1^), *Ruta graveolens* L. (5 mg·mL^−1^), *Vitis vinífera* L. (100 mg·mL^−1^), and the mixed extract, separately. Volumes were completed with methanol HPLC grade, resulting in different concentrations of quercetin and rutin (Cf). The recovery percentages of flavonoid standards (R%) were determinate [30,31].

For robustness determination, six concentrations of mixed sample were analyzed and the analyzer was varied for data comparison [30].

Detection and quantification limits (DL and QL, respectively) were evaluated to determine and quantify the lowest acceptable concentration of quercetin and rutin in extracts [30]. Therefore, rutin and quercetin solution (analytical standards) were prepared containing low concentration, and DL and QL were calculated using Equations (1) and (2).

DL = SD × 3/SC(1)

QL = SD × 10/SC(2)

where SD is standard deviation of intercept with the Y axis of at least three analytical curves constructed, and SC is the slope of analytical curve.

To determine the method selectivity, chromatograms of extracts were compared with analytical standards chromatograms to determine impurities in the extracts [30].

### 2.5. In Vitro Viability Cell Analysis by Neutral Red Uptake (NRU)

#### 2.5.1. Cell Culture Conditions

The immortalized human keratinocyte (HaCaT) cell line was kindly provided by Prof. Ricardo Della Coletta (University of Campinas) and was maintained in RPMI 1640 (Gibco, USA) supplemented with 5% (v/v) fetal bovine serum (FBS, Gibco) and 1% (v/v) penicillin/streptomycin (Nutricell, 1000 U·mL^−1^:1000 g·mL^−1^) in a humidified atmosphere with 5% CO_2_ at 37 °C. For the experiments, HaCaT cells were used between passages 5 to 12.

#### 2.5.2. Samples and Solutions

The four individual extracts (*D. mollis* fava beans, *G. biloba* dry extract, *R. graveolens* leaves, and *V. vinifera* fruit peels) and the mixed extract (5 mg) were initially diluted in DMSO (50 µL), followed by the addition of 950 µL of RPMI 1640/FBS 5% (working solution). Final concentrations (200.0, 125.0, 62.5, 31.25, 15.62, 7.5, and 2.5 μg·mL^−1^) were obtained by serial dilution in RPMI 1640/FBS 5%. Neutral red stoke solution was prepared at 33 μg·mL^−1^ in deionized water. Neutral red work solution was prepared by mixing 1 mL of stoke solution with 79 mL of RPMI-1640 supplemented with 0.5% (v/v) of fetal bovine serum and 1% (v/v) penicillin/streptomycin, followed by 30 min at 37 °C in a water bath and centrifugation (Fanem, São Paulo, Brazil) for 10 min at 1000 rpm.

#### 2.5.3. Cell Viability Assay

The in vitro cell viability assay was performed as described by Stokes et al. [32] and OECD [33]. The HaCaT (4 × 104 cells·mL^−1^), in 96-well plates (100 µL cells·well^−1^), were exposed to the samples’ final concentrations, in triplicate, for 48 h. The final DMSO concentration (≤0.25%) did not affect cell viability. Doxorubicin chloride (0.5 μg·mL^−1^) was used as positive control. After 48 h of exposure, medium was removed and replaced by neutral red work solution (200 μL·well^−1^). Cells were incubated for 3 h, followed by supernatant removal and the addition of ethanol/acetic acid solution (1.0%; v/v) (100 μL·well^−1^). Absorbance values were read at 540 nm using a spectrophotometer (Versamax, Molecular Devices, São Paulo, Brazil). Concentration–response curves for each sample were plotted using GraphPad Prism (version 5.02) software for Windows (GraphPad Software, San Diego, CA, USA).

### 2.6. In Vitro Sun Protection Factor Evaluation

The in vitro sun protection factor was determined by the ultraviolet-visible spectrophotometry method described by Mansur et al. [34]. Spectrophotometric readings were obtained for each extract (100 μg·mL^−1^) at 290–320 nm and SPF values were determined using Equation (3):

SPF = CF × Σ^290^_320_ × EE(λ) × I(λ) × Abs(λ) (3)

where SPF stands for solar protection factor; CF for correction factor; EE_(λ)_ is the erythemogenic effect of wavelength radiation (λ) nm, which was previously calculated by Sayre et al. [35]; I_(λ)_ is the intensity of solar radiation in the wavelength (λ) nm; and Abs_(λ)_ is the spectrophotometry reading of the absorbance of sunscreen solution in the wavelength (λ) nm.

In vitro SPF, critical wavelength (λc), and UVA/UVB rate were assessed by spectral transmittance (Labsphere^®^ UV-2000S Ultraviolet Transmittance Analyzer, Sao Paulo, Brazil) at 250–450 nm, and the instrument bandpass was approximately 1 nm [36].

### 2.7. In Vitro Antioxidant Activity Analysis

For in vitro antioxidant activity, free radicals 1,1-diphenyl-2-picrylhydrazyl (DPPH) and 2,2’-azino-bis(3-ethylbenzilthiazoline-6-sulfonic acid) (ABTS) were used. For the DPPH and ABTS methods, different extract concentrations (75, 125, 200, 400, 500, 750, and 1000 μg·mL^−1^) were used, mixed with an appropriate radical amount and kept in the dark during reaction time [37]. Both assays were performed using quercetin in different concentrations (0.25, 0.5, 1.0, 1.75, 2.5, and 5.0 μg·mL^−1^) as standard, and all assays were performed in triplicate.

Antioxidant evaluation by Oxygen Radical Absorbance Capacity (ORAC) was also determined using fluorescein as the fluorescent molecule and 2,2′-azo-bis(2-amidino propane) dihydrocloreto (AAPH) radical as the oxidant agent. Plates were read in a microplate fluorometer reader (Fluorolog-3 FL3-122, Horiba Jobin Yvon, EUA), and Trolox^TM^ (Sigma-Aldrich—93.0% of purity) was used as standard antioxidant [38,39]. IC_50_ values were calculated using linear regression by Origin software.

### 2.8. Statistical Analysis

All assays were performed in triplicate. Statistical analysis was performed using an ANOVA test (*p* < 0.05) for independent variables, Origin version 8 and Graph Pad Prism (version 5.02) software for Windows (GraphPad Software, San Diego, CA, USA).

## 3. Results and Discussion

After collecting plant material, plants were dried, milled, and presented physical chemical parameters according to Table 1. All samples presented acceptable plant material parameters [28], ensuring quality.

Dry extracts obtained from plants were subjected to identification reaction assays following the guidelines of the Brazilian Pharmacopoeia [28]. All extracts showed rose color in Shinoda and Pew reactions and yellow fluorescence in Taubock reaction. They also exhibited fluorescence under UV light and brown color when subjected to aluminum and ferric chloride, respectively, highlighting the presence of flavonoids in the extracts [40].

The HPLC method was validated for the quantification of flavonoids, for which the parameters of specificity/selectivity, linearity, precision, sensitivity (detection limit and quantification limit), accuracy, and robustness were determined.

The linearity assay was determined for rutin (y = 19862x + 26548; R^2^ = 0.9997; Figure S1) and quercetin (y = 35758x − 58195; R^2^ = 0.9996; Figure S2) analytical curves. For the precision test, the area of the peaks obtained by HPLC of quercetin and rutin standards and these flavonoids in the mixed sample are displayed in Table 2 (intra-day and inter-day assay). Variation coefficients less than 5.0% were obtained, showing the precision of the method [30].

During precision assay, robustness of the method was determined by changing the analyzer. Since a variation coefficient less than 5% was obtained, the quantification method was considered robust.

A recovery assay was performed to determine the accuracy of the method [30]. The percentage of quercetin and rutin recovered (R%) was calculated and results demonstrate the accuracy of the method, as the mean recovery rate was close to 100.0% and variation coefficient less than 5.0% (Table 3). Detection and quantification limits (DL and QL) were calculated from Equations (2) and (3), achieving the following values: DL = 0.86 ± 0.91 μg·mL^−1^ (quercetin) and 9.02 ± 1.12 μg·mL^−1^ (rutin); QL = 2.88 ± 0.92 μg·mL^−1^ (quercetin) and 30.09 ± 1.01 μg·mL^−1^ (rutin). To determined selectivity of the method, the extracts’ chromatograms were compared to standard chromatograms [30,41]. The extracts and mixed sample did not exhibit impurities or other compounds capable to interfere in identification of quercetin and rutin peaks.

**Figure 1:**
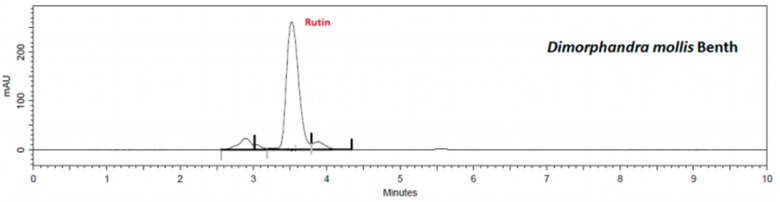
Chromatogram of *D. mollis* Benth extract by HPLC assay.

**Figure 2:**
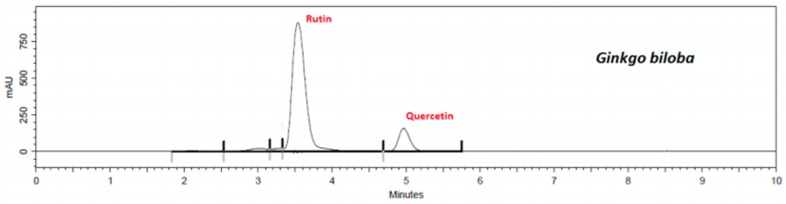
Chromatogram of *G. biloba* extract by HPLC assay.

**Figure 3:**
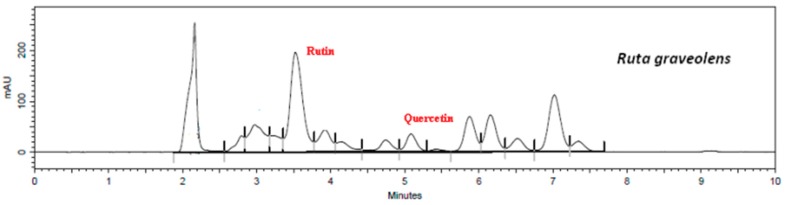
Chromatogram of *R. graveolens* by HPLC assay.

**Figure 4:**
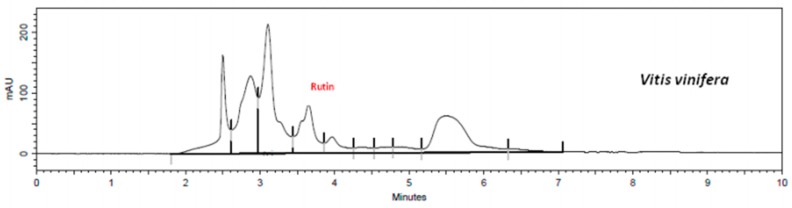
Chromatogram of V. vinifera exract by HPLC assay.

**Figure 5:**
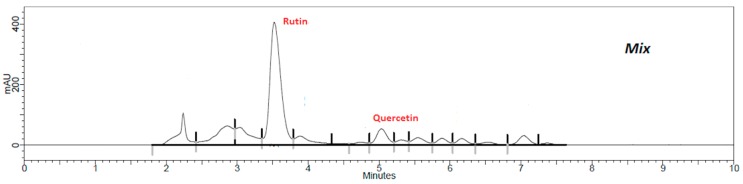
Chromatogram of the mixed sample (1:1:1:1) by HPLC assay.

Using the validated method, quercetin and rutin content in each extract was determined. Flavonoid retention times of samples were similar to standards, i.e., 3.6 min to rutin (Figure S3) and 5.1 min to quercetin (Figure S4). The presence of flavonoids in the extracts and in the mixed sample are shown in Figure 1, Figure 2, Figure 3, Figure 4 and Figure 5.

The results show that *G. biloba* L. extract presented higher quercetin (47.21 ± 1.1 μg·mL^−1^) and rutin (406.84 ± 0.8 μg·mL^−1^) concentrations than *D. mollis* Benth (quercetin = 2.88 ± 0.9 μg·mL^−1^; rutin = 108.17 ± 1.2 μg·mL^−1^), *R. graveolens* L. (quercetin = 11.27 ± 0.7 μg·mL^−1^; rutin = 94.68 ± 1.1 μg·mL^−1^), and *V. vinifera* L. (quercetin = 2.88 ± 0.9 μg·mL^−1^; rutin = 30.09 ± 1.0 μg·mL^−1^). These results are in line with reports from the literature [42,43]. Besides, the mixed sample showed a high concentration of rutin (314.95 ± 0.9 μg·mL^−1^) against the concentration of quercetin of 7.42 ± 0.9 μg·mL^−1^.

**Figure 6:**
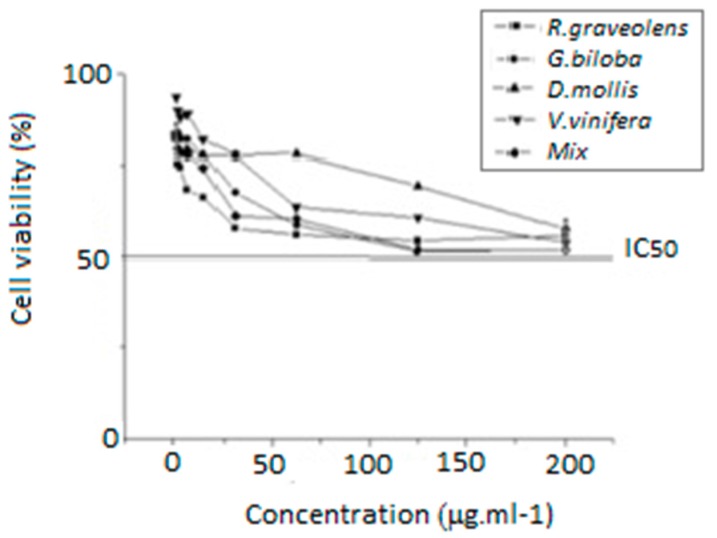
Cell viability curves of samples (*Dimorphandra mollis* Benth, *Ginkgo biloba* L., *Ruta graveolens* L., *Vitis vinífera* L. and mixed (1:1:1:1)) in different concentrations (200.0, 125.0, 62.5, 31.25, 15.62, 7.5, and 2.5 μg·mL^−1^) after 48 h of HaCat cell line exposition by the NRU method.

According to Stokes et al. [32], the evaluation of neutral red uptake is directly proportional to living cell number [33]. In our study, in all tested concentration, the extracts and the mixed sample reduced HaCat cell viability to less than 50% (Figure 6). Thus, the concentration required to reduce by 50% the cell viability (IC_50_) was higher than 200 μg·mL^−1^. We did not test higher concentrations of the selected extracts to avoid the production of artefacts in culture medium, as already described for many phenolic compounds [27].

Spectrophotometry in the ultraviolet region is an adjuvant and preliminary in vitro method to evaluate sun protection factor of compounds, especially from vegetal sources [34,44,45]. Thus, the four extracts (100 μg·mL^−1^) and mixed sample were subjected to spectrophotometric analysis. *D. mollis* Benth presented an SPF value of 5.04 ± 0.2, *G. biloba* L. of 8.31 ± 0.5, *R. graveolens* L. of 7.08 ± 0.4, *V. vinifera* L. of 3.71 ± 0.5, and the mixed sample (1:1:1:1) of 7.72 ± 0.4.

As determined by cell viability assay, both SPF assays were performed in concentration up to 200 μg·mL^−1^ that was determined as the highest non-cytotoxic concentration tested in our work. These preliminary results show that *G. biloba* and *R. graveolens* extracts were the most promising extracts, besides the mixed sample. To confirm the SPF results, all samples were then evaluated using spectral transmittance [46,47]. All individual extracts, the mixed sample, and the positive control (Tinosorb S^TM^) presented absorption in UVA and UVB regions (Table 4) in different ways. While *D. mollis* and *G. biloba* extracts absorbed in the 320–400 nm range, corresponding to UVA radiation, *R. graveolens* and *V. vinifera* extracts absorbed around 310 nm, corresponding to UVB radiation. Moreover, the mixed sample and Tinosorb S^TM^ absorbed in a higher range, configuring protection in UVA and UVB regions [6]. *G. biloba* L. extract, followed by the mixed sample, presented the highest SPF values (Table 4), attributed to the higher flavonoid concentration. Moreover, *D. mollis* Benth and *V. vinifera* L. extracts that showed the lowest quercetin concentration presented low values, corroborating literature data [48].

In addition, according to the literature [16,49,50], the SPF values found in the extracts studied in our work were lower than chemical sun filters, such as Tinosorb S^TM^.

The colorimetric evaluation shows that all individual extracts have flavonoids and they can therefore be considered a promising plant sources to be used as sunscreen. 

Antioxidant activity of phenolic compounds such as flavonoids is widely known, and thus, flavonoids are widely studied as ingredients in cosmetic formulations against early skin aging by scavenging reactive oxygen species produced by sun radiation [4,22,51].

All of the four extracts, together with the mixed sample, were subjected to in vitro antioxidant assays. From the DPPH and ABTS experiments, the results were expressed as the sample concentration required for 50% reduction of the radical concentration, while on ORAC protocol, the ability of scavenger peroxyl radicals was expressed as the equivalent concentration of Trolox [39]. Then, IC_50_ values were calculated, and once again, the best results were seen for *G. biloba* L. extract and the mixed sample (Table 5), and this can be attributed to the higher flavonoid concentration in these samples.

Based on these results, the extracts and the mixed sample presented antioxidant activity against DPPH, ABTS, and AAPH radicals, which is indicative of premature aging protection. Then, considering the samples’ potential as sunscreens, all extracts and mixed samples can be incorporated into a cosmetic formulation, aiming to develop a new sunscreen containing chemical sun filter from plant material.

## 4. Conclusions

This work is the first study about the sun protection action of *Dimorphandra mollis* Benth and *Ruta graveolens* L. All flavonoid-enriched extracts were not cytotoxic, and presented antioxidant activity and sun protection factor, as shown by in vitro methods. The mixed sample composed by the four studied plants presented promising results. The quantification method exhibited linearity, precision, accuracy, robustness, and did not exhibit impurities or other compounds capable of interfering in the identification peak of flavonoids. The mixed sample may be an alternative to treat deleterious effects from exposure to ultraviolet radiation and a promise as a potential sunscreen.

## Figures and Tables

**Table 1:** Physical–chemical parameters of *D. mollis* Benth (dry favas), *G. biloba* L. (dry extract), *R. graveolens* L. (dry leaves), and *V. vinífera* L. (dry peels). Values are presented as an average of three measurements and standard deviation (±SD).

Species	Granulometry (mm)	Density (g·mL^−1^)	pH	Dry Loss (%)	Total Ash (%)	Total Insoluble Ash (%)
*D. mollis* Benth	1.40 ± 1.1	0.27 ± 0.3	5.01 ± 0.8	2.51 ± 1.2	6.98 ± 1.3	1.55 ± 1.1
*G. biloba* L.	0.35 ± 1.0	0.58 ± 0.2	4.86 ± 1.1	4.60 ± 1.4	5.78 ± 1.3	1.76 ± 1.1
*R. graveolens* L.	0.60 ± 1.2	0.91 ± 0.2	5.90 ± 1.1	4.44 ± 1.1	6.76 ± 1.2	1.54 ± 0.9
*V. vinifera* L.	0.60 ± 0.9	0.45 ± 0.2	3.96 ± 1.1	4.83 ± 1.1	6.92 ± 1.4	1.35 ± 1.0

**Table 2:** Intra-day and inter-day precision values for analytical flavonoids standard (quercetin and rutin) and in mixed samples [mix (quercetin) and mix (rutin)]. Values are presented as an average of three measurements and standard deviation (±SD).

Sample	Peak Area		Total Variation Coefficient (%)
	Inter-day	Intra-day	
**Quercetin**	468.63 ± 0.1	469.81 ± 0.1	0.28 ± 0.0
Rutin	269.65 ± 0.1	270.30 ± 0.1	0.89 ± 0.1
Mix (quercetin)	600,236.80 ± 0.1	602,254.30 ± 0.0	1.78 ± 0.0
Mix (rutin)	4,491,918.70 ± 0.0	4,573,531.81 ± 0.1	1.62 ± 0.1

**Table 3:** Recovery assay values referents to quercetin and rutin concentration in extracts. Values are presented as an average of three measurements and standard deviation (± SD).

Extracts	Samples	Standard vol.(Cr) (mL)	Final Coef. of Quercetin (Cf)	Final Coef. of Rutin (Cf)	% quer. rec. (R_q_%)	% rutin rec. (R_r_%)	Var. Coef. R_q._%	Var. Coef. Rr_._%
*D. mollis* Benth	R1	0.75	5.44 ± 0.2	85.39 ± 0.1	102.05 ± 0.1	110.22 ± 0.1	0.30 ± 0.2	3.64 ± 0.2
	R2	1.25	9.91 ± 0.1	140.88 ± 0.1	102.58 ± 0.1	104.73 ± 0,1		
	R3	1.75	14.88 ± 0.1	192.83 ± 0.1	102.61 ± 0.1	102.78 ± 0.2		
*G. biloba* L.	R1	0.75	10.72 ± 0.2	139.22 ± 0.2	102.82 ± 0.1	106.53 ± 0.1	1.79 ± 0.1	1.13 ± 0.2
	R2	1.25	15.43 ± 0.2	204.00 ± 0.1	103.10 ± 0.2	109.10 ± 0.1		
	R3	1.75	18.55 ± 0.1	257.10 ± 0.1	104.98 ± 0.2	110.34 ± 0.1		
*R. graveolens* L.	R1	0.75	6.01 ± 0.1	91.01 ± 0.1	102.44 ± 0.1	103.29 ± 0.1	2.79 ± 0.1	1.02 ± 0.2
	R2	1.25	10.45 ± 0.1	146.39 ± 0.1	103.00 ± 0.2	108.22 ± 0.2		
	R3	1.75	13.97 ± 0.1	196.82 ± 0.2	104.48 ± 0.1	108.65 ± 0.1		
*V. vinifera* L.	R1	0.75	6.21 ± 0.2	81.69 ± 0.2	101.46 ± 0.2	106.02 ± 0.2	2.14 ± 0.2	0.41 ± 0.2
	R2	1.25	10.47 ± 0.2	139.92 ± 0.1	102.19 ± 0.2	102.20 ± 0.2		
	R3	1.75	16.21 ± 0.2	177.16 ± 0.1	101.45 ± 0.2	102.16 ± 0.2		
Mixed	R1	0.75	7.89 ± 0.1	102.03 ± 0.1	100.52 ± 0.1	112.33 ± 0.1	3.55 ± 0.1	0.65 ± 0.1
	R2	1.25	11.59 ± 0.1	169.60 ± 0.2	101.82 ± 0.1	105.23 ± 0.1		
	R3	1.75	16.99 ± 0.1	220.31 ± 0.1	101.43 ± 0.1	106.25 ± 0.2		

**Table 4:** In vitro sun protection factor values of extracts by spectral transmittance.

Extracts (200 μg·mL^−1^)	Critical Wavelength (nm)	UVA *	UVB *	SPF	UVA/UVB Rate
*D. mollis* Benth	398.0 ± 0.1	×		4.96 ± 0.2	0.9 ± 0.0
*G. biloba* L.	388.1 ± 0.0	×		7.06 ± 0.2	0.9 ± 0.0
*R. graveolens* L.	309.0 ± 0.2		×	5.34 ± 0.1	0.9 ± 0.0
*V. vinífera* L.	318.0 ± 0.1		×	3.17 ± 0.2	0.9 ± 0.0
Mixed sample	372.7 ± 0.1	×	×	6.92 ± 0.1	0.8 ± 0.0
Tinosorb S^TM^	369.1 ± 0.1	×	×	21.01 ± 0.2	0.7 ± 0.0

* UVA range equal to 320–400 nm and UVB equal to 280–315 nm.

**Table 5:** In vitro antioxidant evaluation against DPPH, ABTS, and AAPH free radicals of some potential sunscreen natural products. Values are presented as an average of three measurements and standard deviation (±SD).

Extracts	IC_50_—DPPH (μg·mL^−1^)	IC_50_—ABTS (μg·mL^−1^)	IC_50_—AAPH (μg·mL^−1^)
*D. mollis* Benth	174.51 ± 1.1	596.73 ± 1.6	15.43 ± 1.2
*G. biloba* L.	8.12 ± 0.8	109.09 ± 1.0	25.55 ± 0.4
*R. graveolens* L.	281.02 ± 1.0	587.98 ± 0.8	17.87 ± 0.3
*V. vinífera* L.	296.90 ± 1.2	643.13 ± 0.9	16.08 ± 1.3
Mixed sample	28.73 ± 0.7	325.08 ± 0.8	23.79 ± 0.1
Quercetin	1.75 ± 0.4	2.00 ± 1.2	0.97 ± 0.9

## Supplementary Materials

The following are available online at https://www.mdpi.com/2223-7747/8/11/453/s1: Figure S1. Rutin analytical curve; Figure S2. Quercetin analytical curve; Figure S3. Chromatogram of rutin analytical standard; Figure S4. Chromatogram of quercetin analytical standard.
