# Supplementary figures and images for: Evaluation of In Vitro Solar Protection Factor (SPF), Antioxidant Activity, and Cell Viability of Mixed Vegetable Extracts from Dirmophandra mollis Benth, Ginkgo biloba L., Ruta graveolens L., and Vitis vinífera L
Source: Plants (Basel). 2019 Oct 26;8(11):453. doi: 10.3390/plants8110453 (PMC6918343; doi:10.3390/plants8110453)

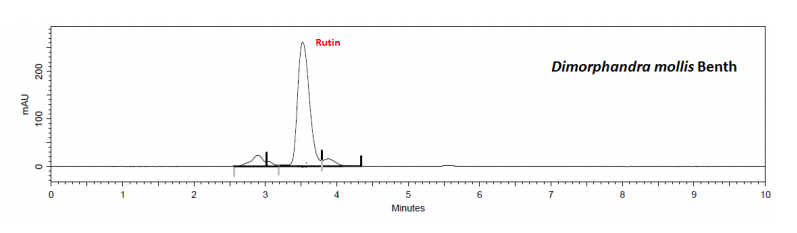

Supplement: Supplementary file 1 [file plants-08-00453-s001.zip › plants-579237_FIGURES/Fig 1.tiff]

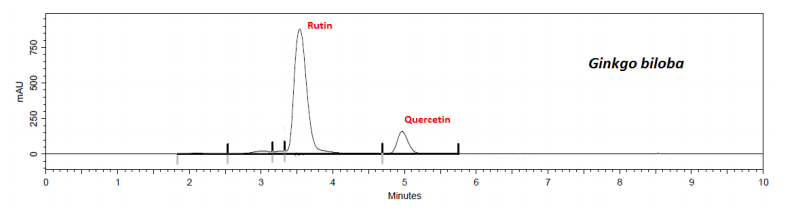

Supplement: Supplementary file 1 [file plants-08-00453-s001.zip › plants-579237_FIGURES/Fig 2.tiff]

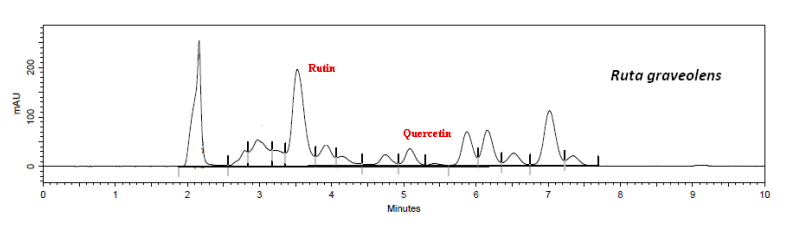

Supplement: Supplementary file 1 [file plants-08-00453-s001.zip › plants-579237_FIGURES/Fig 3.tiff]

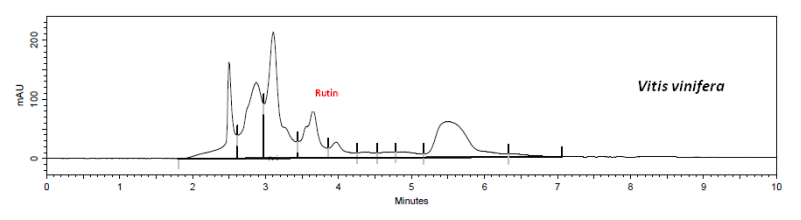

Supplement: Supplementary file 1 [file plants-08-00453-s001.zip › plants-579237_FIGURES/Fig 4.tiff]

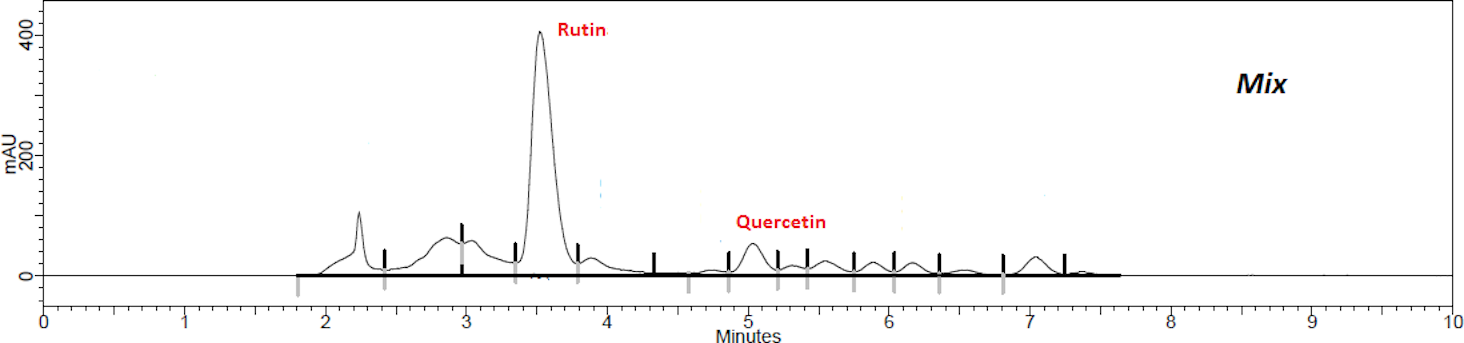

Supplement: Supplementary file 1 [file plants-08-00453-s001.zip › plants-579237_FIGURES/Fig 5.tif]

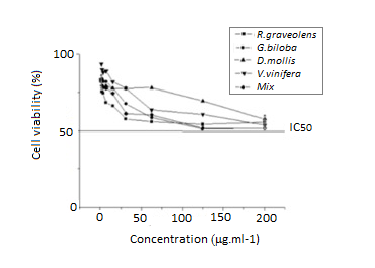

Supplement: Supplementary file 1 [file plants-08-00453-s001.zip › plants-579237_FIGURES/Fig 6.tif]
